# A Textbook of the Practice of Medicine

**Published:** 1910-04

**Authors:** 


**A Textbook of the Practice of Medicine.** By **James M. Anders,** M.D., Ph.D., LL.D., Professor of the Theory and Practice of Medicine and of Clincal Medicine, Medico-Chirurgical College, Philadelphia. Ninth edition. Octavo of 1326 pages, fully illustrated. Philadelphia and London: W. B. Saunders Company. 1909. (Cloth, $5.50; half morocco, $7.00 net prices.)

For the past thirteen years this work has been one of the standard authorities on the subject of which it treats. The present edition, the ninth, has been brought up to the immediate present, without an increase in the size of the volume. The general plan of previous editions, which made the work popular with the medical profession, has been followed in the recent revision. The book deserves, and will no doubt receive, the cordial reception that has been accorded to its predecessors. Anders is recognised as one of the foremost clinicians in the English speaking world and his book is always welcome among teachers, practitioners and students of medicine. J. A. R.

